# Macrobenthic communities of the continental shelf of Heraklion Bay (Crete, Greece): bathymetric distribution and temporal trends

**DOI:** 10.3897/BDJ.13.e174931

**Published:** 2025-12-09

**Authors:** Eleni Koumpaki, Maria Maidanou, Georgios Chatzigeorgiou, Ioannis Rallis, Dimitra Mavraki, Costas Dounas, Christos Arvanitidis, Panayota Koulouri

**Affiliations:** 1 Hellenic Centre for Marine Research, Heraklion, Crete, Greece Hellenic Centre for Marine Research Heraklion, Crete Greece; 2 Artificial Reef Innovative Applications, Heraklion, Crete, Greece Artificial Reef Innovative Applications Heraklion, Crete Greece; 3 LifeWatch ERIC, Seville, Spain LifeWatch ERIC Seville Spain

**Keywords:** macrobenthos, soft-bottom, long-term monitoring, bathymetric distribution, Heraklion Bay, Cretan Sea, Mediterranean

## Abstract

**Background:**

The Gulf of Heraklion is one of the most well-studied coastal marine ecosystems in the eastern Mediterranean. It is an oligotrophic area, exposed to wave action where muddy sediments prevail and its coastal zone is relatively unaffected by major riverine inputs. However, it faces pressures deriving from urbanisation, tourism, small-scale fisheries and climate change. Amongst its biological components, macrobenthos is an important component of soft-bottom habitats of the continental shelf and considered to be an indicator of environmental and human-induced disturbance. Nevertheless, long-term data are limited, thus restricting our understanding of their temporal trends. This is of particular importance for the Mediterranean Sea which is characterised as a hot spot of biodiversity and climate change impacts on its biota.

**New information:**

The macrοbenthic faunal communities were investigated in the continental shelf of Heraklion Bay within the framework of the European funded project entitled MARine Biodiversity and Ecosystem Functioning leading to Ecosystem Services (MARBEFES), with the objective to enhance the understanding of marine biodiversity, ecosystem functioning and the ecosystem services they provide. Additional studies, with the same objectives, were carried under national and local monitoring programmes. Samples were collected from a specific transect of stations, named H2 (10, 20, 30, 40, 50, 100 and 200 m depth), located in the wider marine area of the wastewater treatment plant of Heraklion City. The transect was sampled during three different sampling surveys and periods: June 2010, June 2015 and August 2024.

A total of 30,280 individuals were examined belonging to the taxonomic groups of Annelida, Crustacea, Mollusca, Echinodermata, Nemertea and varia. The identification of the key taxonomic classes (Polychaeta, Bivalvia, Gastropoda, Scaphopoda and Malacostraca) revealed 231 macrobenthic taxa. Out of the 231 taxa, only 30 were common amongst all the three sampling periods, while Polychaeta was the most abundant macrobenthic group in all sampling periods.

## Introduction

Amongst the coastal marine ecosystems of the eastern Mediterranean, Heraklion Bay (Crete, Greece) has been the focus of research for several decades. Its accessibility and proximity to the Hellenic Centre for Marine Research have facilitated multi-disciplinary and cross-domain approaches, implemented by multiple research projects.

Early investigations provided important information on the environmental characteristics of the Bay. It is described as oligotrophic with primary productivity of 80 gC/m²/year (Psarra et al. 2000), characterised by intense wave action and muddy sediments (Chronis et al. 2000). The continental shelf is also considered relatively unaffected by major river and wastewater discharges (Karakassis and Eleftheriou 1997, Koulouri et al. 2005, Koulouri et al. 2006, Poulos et al. 2008, Koukounari et al. 2020).

More recent assessments, however, indicate that this coastal ecosystem now faces increasing pressures from urbanisation, tourism, small-scale fisheries and climate change (Koulouri et al. 2005, Chatzinikolaou and Arvanitidis 2016, Rallis et al. 2022).

Given their ecological importance, macrobenthic communities have been the focus of numerous studies over the years, particularly within soft-bottom habitats, documenting their species composition and spatial distribution (Koutsoubas 1992, Karakassis and Eleftheriou 1997, Plaiti et al. 2000, Tselepides et al. 2000, Koulouri et al. 2006, Koulouri et al. 2013, Tsikopoulou et al. 2019). This growing attention reflects the central role of macrobenthos in benthic ecosystems. Macrobenthic communities are amongst the most sensitive indicators of environmental changes and human impacts on marine habitats and are also key biological quality elements (BQEs) under the Marine Strategy Framework Directive (MSFD, DIRECTIVE 2008/56/EC; European Commission (2008)) and the Water Framework Directive (WFD, 2000/60/EC; European Commission (2000)).

Despite this research background, long-term studies remain scarce, making it difficult to understand the ecological status and changes over time. This issue is particularly critical for the Mediterranean Sea, a region widely recognised for both its high biodiversity and vulnerability to climate change (Coll et al. 2010, Tuel and Eltahir 2020).

The present study focuses on the bathymetric distribution of macrobenthic communities of the continental shelf of Heraklion Bay along with its temporal trends and changes, particularly adjacent to the wastewater treatment plant of Heraklion City. By updating existing datasets and providing new insights into bathymetric and long-term ecological shifts in macrobenthic faunal communities, this research supports both the conservation and protection of marine biodiversity and contributes to the development of effective management strategies for the marine environment of Heraklion Bay.

## General description

### Purpose

**Figure 1.**
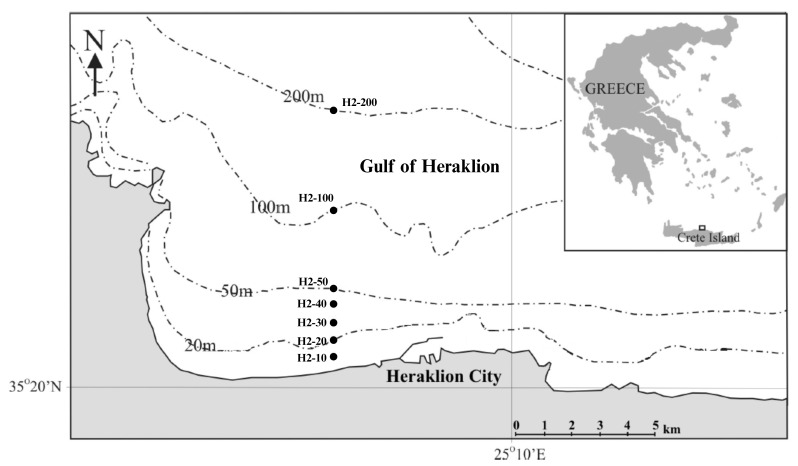
Map of the study area showing transect H2 and its stations located in Heraklion Bay (Crete, Greece).

This study examines taxon composition and abundance of macrobenthic communities along the H2 transect of Heraklion Bay, located in the wider area of the wastewater treatment plant of Heraklion City (Fig. 1). Sampling was carried out during three different sampling surveys and periods — June 2010, June 2015 and August 2024 — at different depths (10, 20, 30, 40, 50, 100 and 200 m) along the soft-bottom continental shelf, as part of three different projects: MARBEFES, monitoring programmes of both the National Strategic Reference Framework 2007-2013 and the Municipal company of water supply and sewage of Heraklion City.

## Project description

### Title

MARBEFES: MARine Biodiversity and Ecosystem Functioning leading to Ecosystem Services; KRIPIS I: Marine Biology, Biotechnology & Aquaculture; DEYAH: Monitoring programme of the wastewater treatment plant of Heraklion City.

### Personnel

Eleni Koumpaki (sampling, taxonomic identification, data management, manuscript writing), Dr Maria Maidanou (sampling, taxonomic identification), Dr Georgios Chatzigeorgiou (sampling, taxonomic identification), Ioannis Rallis (data curation and management), Dimitra Mavraki (data curation and management), Dr Costas Dounas (expedition design and sampling, taxonomic identification), Dr Christos Arvanitidis (taxonomic identification, manuscript editing), Dr Panayota Koulouri (principal investigator and project manager, expedition design and sampling, taxonomic identification, manuscript editing).

### Study area description

H2 transect, Heraklion Bay, Heraklion (Crete, Greece).

### Design description

This study was designed to address the knowledge gaps on the differences of the macrobenthic communities along two scales: a) temporal (three sampling periods) and b) bathymetric (seven depth stations).

### Funding

This study has been financed by the following projects:

1. MARBEFES: Marine Biodiversity and Ecosystem Functioning leading to Ecosystem Services (Grant Agreement no 101060937);
2. Operational Programme of “Competitiveness & Entrepreneurship” of the National Strategic Reference Framework (NSRF 2007-2013) in the framework of the project “Marine Biology, Biotechnology & Aquaculture” (MIS 453278) by Greece and the European Union (European Regional Development Fund);
3. Monitoring programme of the wastewater treatment plant of Heraklion City by the Municipal company of water supply and sewage of Heraklion City.

Data management and publication were supported by LifeWatch Greece and MedOBIS was selected as the open and FAIR repository to host the relevant dataset.

## Sampling methods

### Sampling description

Three replicate sediment samples were collected along the H2 transect, covering depths from 10 m to 200 m, during the period 2010–2024. A Smith McIntyre grab (SMI, sampling surface: 0.1 m²) has been used, operated by the research vessel “PHILIA” of the Hellenic Centre for Marine Research. The position of each station was calculated using a FURUNO SFN-70 satellite navigator and depth was measured by a SIMRAD K-400 echo sounder. The samples have been sieved *in situ* with a 0.5 mm sieve and preserved in 95% ethanol buffered with seawater. Macrobenthic taxa were then sorted in the lab from the sediment and then counted and identified to the lowest possible taxonomic level, where possible, using a stereoscope and a microscope.

### Quality control

All the samples were processed in the same way and the species were cross-validated by experts.

All data were standardised following the FAIR principles (Findable, Accessible, Interoperable and Reusable), ensuring that both metadata and data are machine-actionable and properly linked via persistent identifiers and community standards (Wilkinson et al. 2016). Taxonomic and occurrence information were standardised using the Darwin Core standards, which provide a globally accepted framework for saving and sharing biodiversity data with consistent terminology (Wieczorek et al. 2012). Scientific names were harmonised using the World Register of Marine Species using the Taxon Match Tool (WoRMS Editorial Board 2025). Additional measurements and associated facts were standardised with controlled terms from the NERC Vocabulary Server, enhancing interoperability across marine data repositories (BODC - British Oceanographic Data Centre 2025). Finally, for quality control, the dataset was tested using the LifeWatch & EMODnet Biology Quality Control tool (Perez et al. 2024), which evaluates Darwin Core Archive formats against EMODnet Biology data quality criteria.

## Geographic coverage

### Description

One transect (H2) of seven stations was selected as a study site presented in Fig. 1. The first two stations are located close to the marine area of the wastewater treatment plant of Heraklion City.

The selection criteria for the specific stations were primarily based on the available data from previous different research projects and studies. The geographical coordinates and depths of the sampling stations along the transect H2 of Heraklion Bay are listed in Table 1.

## Taxonomic coverage

### Description

Temporal changes in the composition of the macrobenthic community are reflected in the abundance of the major taxonomic groups (Annelida, Crustacea, Echinodermata, Mollusca, Nemertea, varia) recorded during the three sampling periods (Table 2). The varia group includes specimens that could not be reliably assigned to any of the taxonomic groups mentioned above due to fragmentation or physical damage.

The data paper presents information on the key macrobenthic taxa and their abundance found in the stations of the three sampling periods, belonging to the following five main classes: Bivalvia, Gastropoda, Malacostraca, Polychaeta and Scaphopoda (Suppl. material 1). For consistent comparison across years, the supplementary material includes only the taxa identified to species or family level in all three sampling periods, representing these six classes. Average densities from the three replicate samples, are expressed in individuals per square metre (ind./m²).

The highest number of taxa and individuals was recorded in June 2015 (145 taxa, average total 4,477 individuals) and within the range of 10–30 m depth (30-75 taxa, average range 447-1,817 individuals) for all sampling periods.

**Figure 2.**
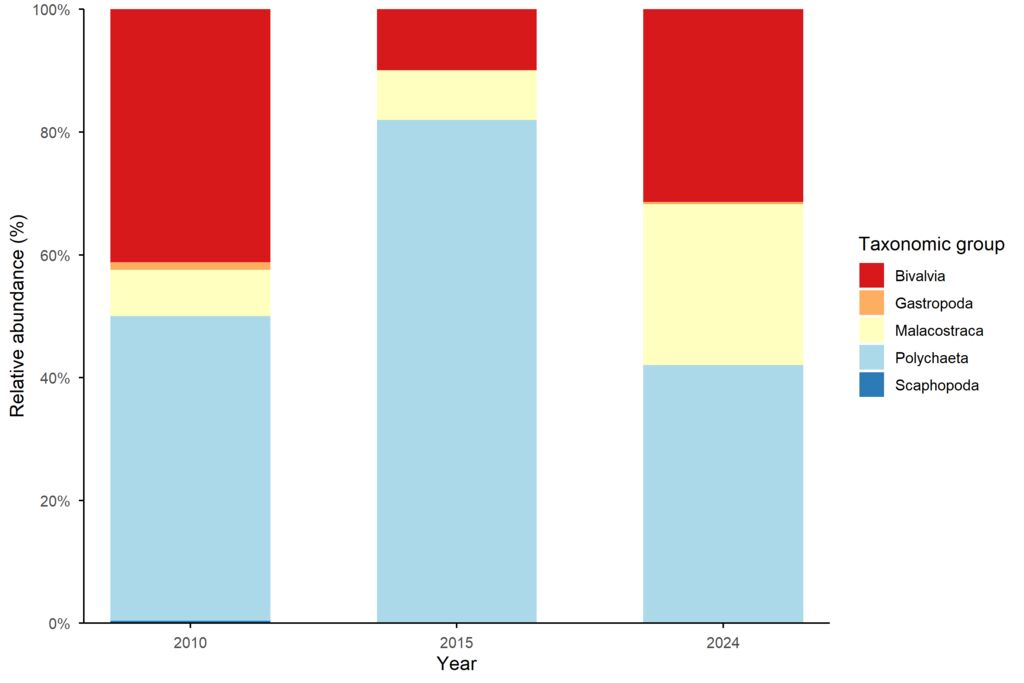
Temporal distribution of the five classes (Bivalvia, Gastropoda, Malacostraca, Polychaeta and Scaphopoda) during the three sampling periods.

Polychaeta were the most abundant class for all the sampling periods, followed by Bivalvia and Malacostraca, while Gastropoda and Scaphopoda were represented in very low percentages (Fig. 2).

## Temporal coverage

**Data range:** 2010-6-18 – 2024-8-05.

## Collection data

### Collection name

Bathymetric distribution and temporal trends of macrobenthic communities of the continental shelf of Heraklion Bay (Crete-Greece).

### Specimen preservation method

95% ethanol buffered with water.

### Curatorial unit

Hellenic Centre for Marine Research (HCMR), Institute of Marine Biology, Biotechnology and Aquaculture (IMBBC), Heraklion, Crete, Greece.

## Usage licence

### Usage licence

Other

### IP rights notes

Creative Commons Attribution 4.0 International (CC-BY-4.0).

## Data resources

### Data package title

Bathymetric distribution and temporal trends of macrobenthic communities of the continental shelf of Heraklion Bay (Crete-Greece)

### Resource link

https://doi.org/10.25607/s8k2pg

### Alternative identifiers

http://ipt.medobis.eu/resource?r=macrosoft&amp;v=1.0

### Number of data sets

1

### Data set 1.

#### Data set name

Bathymetric distribution and temporal trends of macrobenthic communities of the continental shelf of Heraklion Bay (Crete-Greece)

#### Data format

Darwin Core Archive

#### Character set

UTF-8

#### Description

The dataset is available via the MedOBIS (Mediterranean node of Ocean Biodiversity Information System), Integrated Publishing Toolkit (IPT) which has been established through the LifeWatchGreece Research Infrastructure and is hosted in the Institute of Marine Biology, Biotechnology and Aquaculture (IMBBC) of the Hellenic Centre for Marine Research (HCMR). The data are also harvested by and made available through global data repositories, such as the Ocean Biodiversity Information System (OBIS). The dataset is available as a DarwinCoreArchive and all fields are mapped to DarwinCore terms (Koumpaki et al. 2025).

This publication refers to selected data (H2 transect) of the most recent version of the dataset available through the IPT server or MedOBIS.

The current publication refers to the "occurrence" source file (.txt file) that is associated with the particular dataset. While the full dataset reports abundances per 0.1 m² and includes additional replicates, transects and taxa, this data paper focuses specifically on transect H2 across the three sampling periods. To ensure consistent temporal comparisons of biodiversity, only the macrobenthic classes for which taxa were identified to species or family level in all samplng periods are included (Bivalvia, Gastropoda, Malacostraca, Polychaeta, Scaphopoda).

Additional details about the sampling events can be found in the "event" source file (.txt file) associated with the same dataset.

**Data set 1.** 

Column label	Column description
id (Event core, Occurrence)	A unique identifier for the record within the dataset or collection, auto-incrementing number automatically added by the system, different between event core and occurrence.
type (Event core)	The nature or genre of the resource - here expedition.
language (Event core)	The language of the resource - here English.
licence (Event core)	A legal document giving official permission to do something with the resource - here CC BY 4.0.
rightsHolder (Event core)	The organisation owning or managing rights over the resource - here Hellenic Centre for Marine Research (HCMR).
institutionID (Event core)	An identifier for the institution having custody of the object(s) or information referred to in the record - here https://ror.org/038kffh84.
institutionCode (Event core, Occurrence)	The acronym in use by the institution having custody of the object(s) or information referred to in the record - here HCMR - IMBBC.
eventID (Event core, Occurrence)	An identifier specific to the dataset associated with each event.
parentEventID (Event core)	An identifier that describes the station, year and transect for each event.
eventDate (Event core)	The date during which the event occurred.
habitat (Event core)	A category of the habitat in which the event occurred - here sediment type according to EUNIS habitat classification.
samplingProtocol (Event core)	The descriptions of the methods used during a sampling event - here Smith McIntyre grab (SMI, sampling surface: 0.1 m²).
sampleSizeValue (Event core)	A numeric value for a measurement of the sample size – here 0.1 m² (surface area of sampler).
sampleSizeUnit (Event core)	The unit of measurement of the sample size – here representing the surface area of the sampler in m².
samplingEffort (Event core)	The amount of effort expended during an event - here expressed in replicates.
eventRemarks (Event core)	Comments or notes about the event - here the name as the replicate.
locationID (Event core)	An identifier for the set of locality information - here based on the Marine Gazetteer Placedetails.
continent (Event core)	The name of the continent in which the event occurred - here Europe.
country (Event core)	The name of the country or major administrative unit in which the event occurred - here Greece.
countryCode (Event core)	The standard code for the country in which the event occured - here GR.
locality (Event core)	The specific description of the place - here Gulf of Heraklion.
minimumDepthInMetres (Event core)	The lesser depth of a range of depth below the local surface, in metres.
maximumDepthInMetres (Event core)	The greater depth of a range of depth below the local surface, in metres.
locationRemarks (Event core)	Name of the station where each event occurred.
decimalLatitude (Event core)	The geographic latitude of the geographic centre of the event (in decimal degrees, WGS84).
decimalLongitude (Event core)	The geographic longitude of the geographic centre of the event (in decimal degrees, WGS84).
geodeticDatum (Event core)	The ellipsoid, geodetic datum or spatial reference system (SRS) upon which the geographic coordinates are based - here WGS84.
georeferenceProtocol (Event core)	A description or reference to the methods used to determine the coordinates - here GPS.
datasetName (Occurrence)	The name identifying the dataset from which the record was derived.
ownerInstitutionCode (Occurrence)	The acronym in use by the institution having ownership of the object(s) referred to in the dataset - here HCMR - IMBBC.
basisOfRecord (Occurrence)	The specific nature of the data record - here observation.
occurrenceID (Occurrence)	Unique identifier for each occurrence record.
individualCount (Occurrence)	The number of individuals of the same taxon present in each sample.
occurrenceStatus (Occurrence)	A statement about the presence or absence of an occurrence - here only presence.
disposition (Occurrence)	The current state of the sample - here in collection.
verbatimIdentification (Occurrence)	A string representing the taxonomic identification as it appeared in the original record.
scientificNameID (Occurrence)	An identifier for the nomenclatural (not taxonomic) details of a scientific name.
scientificName (Occurrence)	The scientific name.
taxonRank (Occurrence)	The taxonomic rank of the most specific name in scientificName - here species, genus, family.
verbatimTaxonRank (Occurrence)	The taxonomic rank of the most specific name as it appears in the original record - in case of a genus or family level, it indicates the different sp.
scientificNameAuthorship (Occurrence)	The authorship information for scientificName.
taxonRemarks (Occurrence)	Notes about the taxon.
kingdom (Occurrence)	The full scientific name of the kingdom in which the taxon is classified - here Animalia.
phylum (Occurrence)	The full scientific name of the phylum or division in which the taxon is classified.
class (Occurrence)	The full scientific name of the class in which the taxon is classified.
order (Occurrence)	The full scientific name of the order in which the taxon is classified.
family (Occurrence)	The full scientific name of the family in which the taxon is classified.
genus (Occurrence)	The full scientific name of the genus.
subgenus (Occurrence)	The full scientific name of the subgenus in which the taxon is classified.
specificEpithet (Occurrence)	The name of the first or species epithet of the scientificName.
infraspecificEpithet (Occurrence)	The name of the lowest or terminal infraspecific epithet of the dwc:scientificName, excluding any rank designation.

## Supplementary Material

C7063D47-A72F-50F9-BA56-8B02481FD30E 10.3897/BDJ.13.e174931.suppl1 Supplementary material 1 Macrobenthic species identified in the samples collected Data type Occurrences Brief description Macrobenthic species identified in the samples collected in the three sampling periods from stations located at depths between 10 and 200 m. File: oo_1407655.pdf https://binary.pensoft.net/file/1407655 Eleni Koumpaki, Maria Maidanou, Georgios Chatzigeorgiou, Costas Dounas, Christos Arvanitidis, Panayota Koulouri

## Figures and Tables

**Table 1.** Geographical coordinates (decimal degrees) and depths (m) of the sampling stations along transect H2 of Heraklion Bay.

**Station**	**Depth (m)**	**Coordinates**
H2-10	10	35.346472° N	25.114750° E
H2-20	20	35.351667° N	25.114194° E
H2-30	30	35.357306° N	25.113722° E
H2-40	40	35.359806° N	25.110639° E
H2-50	50	35.363306° N	25.109556° E
H2-100	100	35.382556° N	25.108306° E
H2-200	200	35.418194° N	25.113139° E

**Table 2.** Total abundance (ind./m²) of macrobenthic taxonomic groups recorded in the sampling periods of 2010, 2015 and 2024.

**Taxonomic group**	**2010**	**2015**	**2024**
** Annelida **			
Polychaeta	5400	10880	1220
Sipuncula	410	1730	60
** Crustacea **			
Amphipoda	430	350	230
Cumacea	20	110	10
Decapoda	330	600	360
Isopoda	0	0	40
Mysida	40	0	10
Tanaidacea	0	10	110
** Echinodermata **	150	240	360
** Mollusca **			
Bivalvia	4480	1320	910
Gastropoda	130	0	10
Scaphopoda	40	10	0
** Nemertea **	90	140	20
**varia**	30	0	0
