# Supplementary material for: Macrobenthic communities of the continental shelf of Heraklion Bay (Crete, Greece): bathymetric distribution and temporal trends
Source: Biodivers Data J. 2025 Dec 9;13:e174931. doi: 10.3897/BDJ.13.e174931 (PMC12709197; doi:10.3897/BDJ.13.e174931)
Supplement: Supplementary material 1 — Macrobenthic species identified in the samples collected [file bdj-13-e174931-s001.pdf]

Table 1. Macrobenthic species identified in the samples collected in June 2010 from stations located at depths between 10 and 200 m. The values of their average densities from the three replicate samples, are expressed in individuals per square meter (ind./m²).

Species	10 m	20 m	30 m	40 m	50 m	100 m	200 m
**Bivalvia**							
Abra alba (W. Wood, 1802)					3.33		3.33
Abra prismatica (Montagu, 1808)					10.00		
Azorinus chamasolen (da Costa, 1778)			20.00	3.33			
Cuspidaria cuspidata (Olivi, 1792)				3.33			
Donax trunculus Linnaeus, 1758	3.33						
Fabulina fabula (Gmelin, 1791)	46.67						
Loripes orbiculatus Poli, 1795	83.33	30.00					
Loripinus fragilis (Philippi, 1836)	63.33	530.00	13.33	3.33			
Lucinella divaricata (Linnaeus, 1758)	16.67	213.33	13.33				
Moerella donacina (Linnaeus, 1758)		100.00		6.67			
Moerella pulchella (Lamarck, 1818)			13.33				
Myrtea spinifera (Montagu, 1803)	3.33		3.33	3.33			
Nucula mariae Nolf, 2005	3.33	23.33	13.33	6.67			
Nucula nitidosa Winckworth, 1930				3.33			
Pitar rudis (Poli, 1795)		3.33					
Saccella commutata (Philippi, 1844)						3.33	
Thyasira flexuosa (Montagu, 1803)		180.00	30.00	16.67		3.33	
Varicorbula gibba (Olivi, 1792)		3.33	3.33		10.00		
**Gastropoda**							
Ascobulla fragilis (Jeffreys, 1856)		36.67					
Neverita josephinia Risso, 1826	3.33						
Philine aperta (Linnaeus, 1767)		3.33					
**Malacostraca**							
Amphipoda							
Apherusa bispinosa (Spence Bate, 1857)	10.00	3.33	10.00				
Caprella acanthifera Leach, 1814	10.00						
Ericthonius punctatus (Spence Bate, 1857)	3.33	10.00					
Hippolyte inermis Leach, 1816	3.33						
Hippolyte leptocerus (Heller, 1863)	3.33						

Iphimedia minuta G.O. Sars, 1883		3.33					
Iphimedia obesa Rathke, 1843	22.00						
Leucothoe incisa Robertson, 1892	10.00	6.67					
Nototropis guttatus (A. Costa in Hope, 1851)	10.00		3.33				
Perioculodes longimanus (Spence Bate & Westwood, 1868)	10.00	3.33					
Phtisica marina Slabber, 1769	20.00		3.33	10.00			3.33
Pseudolirius kroyeri (Haller, 1879)	3.33		3.33				
Synchelidium haplocheles (Grube, 1864)			3.33				
Tryphosa nana (Krøyer, 1846)	3.33						
Westwoodilla rectirostris (Della Valle, 1893)				3.33			
<u>Cumacea</u>							
Iphinoe rhodaniensis Ledoyer, 1965	3.33			3.33			
<u>Decapoda</u>							
Alpheus glaber (Olivi, 1792)					3.33		
Athanas nitescens (Leach, 1814)		3.33					
Callianassa subterranea (Montagu, 1808)			6.67				
Cestopagurus timidus (Roux, 1830)			3.33				
Eualus cranchii (Leach, 1817)			3.33				
Gourretia denticulata (Lutze, 1937)	13.33	3.33	10.00				
Pagurus bernhardus (Linnaeus, 1758)			3.33				
Pagurus cuanensis Bell, 1845		10.00					
Pagurus prideaux Leach, 1815		6.67					
Philocheras trispinosus (Hailstone in Hailstone & Westwood, 1835)	6.67						
Polybius maculatus (Risso, 1827)			3.33				
Processa macrophthalma Nouvel & Holthuis, 1957		3.33	16.67				
Upogebia deltaura (Leach, 1816)		3.33					
<u>Mysida</u>							
Haplostylus lobatus (Nouvel, 1951)	3.33	3.33	3.33		3.33		
**Polychaeta**							
Abyssoninoe bidentata D'Alessandro, Cosentino, Giacobbe, Andaloro & Romeo, 2014	3.33						
Ampharete acutifrons (Grube, 1860)		3.33					

Aonides oxycephala (Sars, 1862)	70.00	53.33	20.00	13.33			
*Aphelochaeta marion*i (Saint-Joseph, 1894)		26.67					
Aponuphis brementi (Fauvel, 1916)	10.00	13.33	20.00	16.67	3.33		
Aricidea (Acmira) simonae Laubier & Ramos, 1974						3.33	
Capitella capitata (Fabricius, 1780)	10.00						
Capitella minima Langerhans, 1880	60.00	13.33	6.67				
Chaetozone sp.		3.33	3.33	3.33		6.67	
Chaetozone zetlandica McIntosh, 1911	13.33	23.33		6.67	3.33		
Cirrophorus branchiatus Ehlers, 1908	56.67	26.67	3.33	3.33	3.33		
Composetia costae (Grube, 1840)				6.67			
Dialychone acustica Claparède, 1868		3.33					
Dialychone dunerificta (Tovar-Hernández, Licciano, Giangrande, 2007)	3.33	10.00					
Dialychone usticensis (Giangrande, Licciano & Castriota, 2006)	3.33						
Diplocirrus capensis Day, 1961		10.00					
Drilonereis filum (Claparède, 1868)		3.33		10.00	13.33		
Euarche tubifex Ehlers, 1887							3.33
Euclymene lombricoides (Quatrefages, 1866)		6.67					
Euclymene oerstedii (Claparède, 1863)	6.67	36.67	3.33				
Euclymene palermitana (Grube, 1840)		3.33					
Eunicidae sp.1	6.67	10.00	3.33				
Fimbriosthenelais minor (Pruvot & Racovitza, 1895)		6.67					
Glycera alba (O.F. Müller, 1776)	6.67						
Glycera fallax Quatrefages, 1850		10.00		3.33	10.00	6.67	
Glycera sp.1				3.33			
Glycera unicornis Lamarck, 1818		3.33				3.33	
Goniadidae sp.					3.33		
Harmothoe antilopes McIntosh, 1876			3.33				
Harmothoe sp.			3.33				
Hesionides sp.		3.33					
Heteromastus filiformis (Claparède, 1864)	6.67						
Kirkegaardia heterochaeta (Laubier, 1961)	6.67	36.67		10.00	6.67	20.00	3.33
Leiochone leiopygos (Grube, 1860)	23.33	16.67	3.33	6.67	6.67		
Lumbrineris latreilli Audouin & Milne Edwards, 1833		23.33	6.67	10.00	3.33	3.33	
Lysidice unicornis (Grube, 1840)		13.33	13.33	6.67			
Lysilla loveni Malmgren, 1866		3.33					
Macroclymene santanderensis (Rioja, 1917)		23.33					

Magelona filiformis Wilson, 1959	6.67	3.33					
Maldane sp.							3.33
Mediomastus capensis Day, 1961		10.00	6.67	6.67	6.67		
Mediomastus fragilis Rasmussen, 1973	3.33	20.00	3.33	23.33	3.33		
Melinna cristata (M. Sars, 1851)		6.67		3.33	3.33		
Metasychis gotoi (Izuka, 1902)						3.33	
Micronephthys longicornis (Perejaslavtseva, 1891)	33.33	43.33			6.67		
Microspio mecznikowiana (Claparède, 1869)			20.00				
Mysta siphodonta (Delle Chiaje, 1830)		3.33					
Nephtys hombergii Savigny in Lamarck, 1818	10.00	3.33					
Nephtys kersivalensis McIntosh, 1908		3.33	6.67				
Nephtys longosetosa Örsted, 1842						6.67	
Nepthys sp.1				3.33			
Nereis lamellosa Ehlers, 1868					3.33		
Notomastus formianus Eisig, 1887		40.00	3.33				
Notomastus latericeus Sars, 1851	3.33						
Onuphis pancerii Claparède, 1868	10.00	6.67				3.33	
Palola siciliensis (Grube, 1840)		3.33					
Paralacydonia paradoxa Fauvel, 1913			3.33		6.67		
Paraonidae sp.			3.33		3.33		3.33
Paraonides myriamae Katzmann & Laubier, 1975	36.67	20.00					
Paucibranchia bellii (Audouin & Milne Edwards, 1833)		3.33		6.67			
Petaloproctus terricolus Quatrefages, 1866			3.33		3.33		
Pilargis verrucosa Saint-Joseph, 1899					3.33		
Pista cristata (Müller, 1776)		10.00	10.00				
Platynereis coccinea (Delle Chiaje, 1822)	3.33						
Poecilochaetus serpens Allen, 1904		3.33					
Polycirrus idex Lavesque, Hutchings, Daffe & Londoño-Mesa, 2020		3.33					
Polynoidae sp.1				3.33			
Praxillella affinis (M. Sars in G.O. Sars, 1872)		3.33					
Praxillella gracilis (M. Sars, 1861)		16.67				3.33	
Pseudoleiocapitella fauveli Harmelin, 1964		6.67	20.00	13.33			3.33
Rhodine loveni Malmgren, 1865		6.67	3.33	6.67		13.33	
Schistomeringos neglecta (Fauvel, 1923)		3.33					
Scolelepis (Scolelepis) squamata (O.F. Muller, 1806)	3.33						
Scoloplos armiger (Müller, 1776)	173.33						

Sigalion mathildae Audouin & Milne Edwards, 1832	3.33						
Sigalion squamosus Delle Chiaje, 1830	13.33	3.33					
Spionidae sp. 1		3.33					
Spionidae sp. 2	10.00						
Streblosoma bairdi (Malmgren, 1866)			6.67	3.33			
Streblospio shrubsolii (Buchanan, 1890)	43.33	3.33					
Syllis garciai (Campoy, 1982)		3.33					
Syllis parapari San Martín & López, 2000		3.33					
Vermiliopsis sp.					3.33		
**Scaphopoda**							
Antalis dentalis (Linnaeus, 1758)							3.33
Fustiaria rubescens (Deshayes, 1825)		10.00					

Table 2. Macrobenthic species identified in the samples collected in June 2015 from stations located at depths between 10 and 200 m. The values of their average densities from the three replicate samples, are expressed in individuals per square meter (ind./m²).

Species	10 m	20 m	30 m	40 m	50 m	100 m	200 m
**Bivalvia**							
Abra alba (W. Wood, 1802)						6.67	3.33
Abra prismatica (Montagu, 1808)					3.33		
Azorinus chamasolen (da Costa, 1778)				6.67			
Chamelea striatula (da Costa, 1778)	6.67						
Donax trunculus Linnaeus, 1758	3.33						
*Dosinia lupinu*s (Linnaeus, 1758)	6.67						
Ensis ensis (Linnaeus, 1758)	3.33						
*Fabulina fabul*a (Gmelin, 1791)	33.33						
Glans trapezia (Linnaeus, 1767)			3.33				
Gouldia minima (Montagu, 1803)			3.33				
*Loripes orbiculatu*s Poli, 1795	146.67						
Loripinus fragilis (R. A. Philippi, 1836)			13.33	10.00			
Lucinella divaricata (Linnaeus, 1758)	46.67	10.00					
Moerella pulchella (Lamarck, 1818)		10.00	23.33	6.67			
Myrtea spinifera (Montagu, 1803)				3.33			
Nucula mariae Nolf, 2005					6.67		
Nucula nitidosa Winckworth, 1930				3.33	10.00		

*Papillicardium minimu*m (R. A. Philippi, 1836)	3.33						
*Parvicardium pinnulatu*m (Conrad, 1831)			3.33				
Pitar rudis (Poli, 1795)			6.67				
Pronucula tenuis A. W. B. Powell, 1927					3.33		3.33
Saccella commutata (R. A. Philippi, 1844)				3.33	6.67	3.33	6.67
Thracia phaseolina (Lamarck, 1818)	3.33	3.33					
Thyasira flexuosa (Montagu, 1803)					26.67	3.33	
**Malacostraca**							
<u>Amphipoda</u>							
Ampelisca sp. 1	46.67	33.33			6.67		
Bathyporeia sp.	6.67						
Iphimedia obesa Rathke, 1843			6.67				
Leucothoe sp.			3.33				
Microdeutopus sp.					3.33		
Perioculodes longimanus (Spence Bate & Westwood, 1868)	6.67						
Westwoodilla sp.		3.33					
<u>Cumacea</u>							
Iphinoe serrata Norman, 1867	16.67				20.00		
<u>Decapoda</u>							
*Anapagurus bicornige*r A. Milne-Edwards & Bouvier, 1892			3.33	3.33			
Dardanus arrosor (Herbst, 1796)			3.33				
Diogenes pugilator (Roux, 1829)	3.33						
Ebalia deshayesi Lucas, 1846		3.33					
Ethusa mascarone (Herbst, 1785)			3.33				
Galathea intermedia Lilljeborg, 1851			6.67				
*Gourretia denticulat*a (Lutze, 1937)	120.00		13.33	3.33			
Pagurus anachoretus Risso, 1827			6.67				
*Pagurus bernhardu*s (Linnaeus, 1758)	3.33						
*Pagurus cuanensi*s Bell, 1845			3.33				
Pagurus sp.1			6.67				
Philocheras monacanthus (Holthuis, 1961)	3.33						
Processa macrophthalma Nouvel & Holthuis, 1957	3.33	3.33	3.33		3.33		

<u>Tanaidacea</u>							
Leptochelia sp.					3.33		
**Polychaeta**							
Abyssoninoe bidentata D'Alessandro, Cosentino, Giacobbe, Andaloro & Romeo, 2014		10.00	56.67	33.33	30.00	3.33	
Ampharete acutifrons (Grube, 1860)				3.33			
Amphicorina armandi (Claparède, 1864)	6.67	3.33					
Aonides oxycephala (Sars, 1862)	40.00	6.67	23.33	13.33			
Aphelochaeta marioni (Saint-Joseph, 1894)		53.33	83.33	63.33	123.33	20.00	
Aponuphis brementi (Fauvel, 1916)	3.33	30.00	60.00	63.33	23.33		
Aricidea (Acmira) simonae Laubier & Ramos, 1974	6.67		16.67	10.00			
Armandia cirrhosa Filippi, 1861		3.33					
Axiothella constricta (Claparède, 1868)	3.33			3.33			
Capitella capitata (Fabricius, 1780)		20.00	36.67	23.33	13.33		
Capitella minima Langerhans, 1880		6.67		3.33			
Capitella sp. 1			3.33				
Capitella sp. 2					3.33		
Capitellidae sp. 1			6.67	6.67			
Capitellidae sp. 2				3.33			
Caulleriella cabbsi Pocklington & Coates, 2010			6.67	3.33			
*Ceratonereis (Composetia) vittat*a Langerhans, 1884				3.33			
Chaetozone sp.			6.67	13.33	20.00		
Chaetozone zetlandica McIntosh, 1911	6.67		30.00	23.33	20.00		
Cirrophorus branchiatus Ehlers, 1908		46.67	26.67	26.67	16.67	6.67	13.33
Cirrophorus furcatus (Hartman, 1957)					6.67		
Cossura soyeri Laubier, 1964			3.33	20.00	40.00	10.00	
Dialychone acustica Claparède, 1868			3.33				
Dialychone dunerificta (Tovar-Hernández, Licciano, Giangrande, 2007)		10.00			3.33		
Diplocirrus sp.						3.33	
Ditrupa arietina (O. F. Müller, 1776)		10.00		10.00			
Drilonereis filum (Claparède, 1868)		13.33	43.33	26.67	6.67		
Euclymene oerstedii (Claparède, 1863)		6.67	36.67	50.00	3.33		
Euclymene palermitana (Grube, 1840)						3.33	
Eunicidae sp.2			13.33	3.33			
Glycera alba (O.F. Müller, 1776)	20.00	13.33		10.00	3.33	10.00	6.67
Glycera fallax Quatrefages, 1850	6.67	10.00	10.00	6.67			

Glycera sp.2				3.33			
Glycera unicornis Lamarck, 1818	13.33			3.33	6.67	3.33	
Glyceridae sp.1			3.33		3.33		
Goniada maculata Örsted, 1843		10.00			6.67		
Harmothoe antilopes McIntosh, 1876			3.33				
Heteromastus filiformis (Claparède, 1864)		3.33	3.33				
Kirkegaardia heterochaeta (Laubier, 1961)	6.67	3.33	136.67	63.33	123.33	3.33	
Laonice sp.				6.67		6.67	3.33
Leiocapitella dollfusi (Fauvel, 1936)			3.33				
Leiochone leiopygos (Grube, 1860)	3.33		16.67				
Lumbrineridae sp.1			3.33				
Lumbrineris latreilli Audouin & Milne Edwards, 1833		3.33	30.00	13.33	86.67	3.33	6.67
Lysidice unicornis (Grube, 1840)		13.33	66.67				
Macroclymene santanderensis (Rioja, 1917)		6.67	6.67	13.33			
Magelona alleni Wilson, 1958		6.67	6.67	10.00	3.33		
Magelona filiformis Wilson, 1959				3.33			
Magelona minuta Eliason, 1962			3.33	3.33	10.00		
Malmgrenia castanea McIntosh, 1876					3.33		
Mediomastus capensis Day, 1961		6.67	6.67	3.33			
Mediomastus fragilis Rasmussen, 1973	10.00	36.67	50.00	23.33	20.00		
Melinna cristata (M. Sars, 1851)			43.33	26.67	3.33		
Metasychis gotoi (Izuka, 1902)						6.67	
Micronephthys longicornis (Perejaslavtseva, 1891)	6.67	30.00	3.33	6.67			
Mysta barbata Malmgren, 1865			3.33		3.33		
Mysta picta (Quatrefages, 1866)		3.33					
Nephtys hombergii Savigny in Lamarck, 1818	20.00	3.33					
Nephtys incisa Malmgren, 1865					6.67	3.33	
Nephtys kersivalensis McIntosh, 1908		63.33					
Nephtys sp.2			3.33				
Nereididae sp.		3.33	3.33				
Nereis lamellosa Ehlers, 1868			10.00	6.67			
Nicomache lumbricalis (Fabricius, 1780)		3.33	10.00	20.00			
Notomastus formianus Eisig, 1887	6.67	13.33	20.00	20.00	6.67	3.33	
Notomastus latericeus Sars, 1851	3.33	16.67	13.33	3.33			
Odontosyllis ctenostoma Claparède, 1868			3.33				
Onuphis pancerii Claparède, 1868	3.33		3.33			3.33	

Ophelina sp.						3.33	
Palola siciliensis (Grube, 1840)					3.33		
Paradoneis lyra (Southern, 1914)	26.67	16.67	80.00	66.67			
Paralacydonia paradoxa Fauvel, 1913						6.67	
Paucibranchia bellii (Audouin & Milne Edwards, 1833)			10.00	3.33			6.67
Petaloproctus terricolus Quatrefages, 1866				3.33			
Pista cristata (Müller, 1776)			3.33				
Platynereis dumerilii (Audouin & Milne Edwards, 1833)		3.33	3.33				
Poecilochaetus serpens Allen, 1904			6.67		10.00	3.33	
Polycirrus idex Lavesque, Hutchings, Daffe & Londoño-Mesa, 2020				3.33			
Polynoidae sp.2						3.33	
Praxillella affinis (M. Sars in G.O. Sars, 1872)							3.33
Pseudoleiocapitella fauveli Harmelin, 1964	6.67	3.33	6.67	10.00	3.33		
Rhodine loveni Malmgren, 1865		16.67	43.33	60.00	6.67	3.33	
Sabellidae sp.1		3.33					
Schistomeringos neglecta (Fauvel, 1923)			3.33	3.33			
Schistomeringos rudolphi (Delle Chiaje, 1828)	6.67						
Scoloplos armiger (Müller, 1776)	150.00	10.00	3.33			6.67	
Sigalion mathildae Audouin & Milne Edwards, 1832	20.00	3.33					
Sigalion sp.1		3.33					
Sigalion squamosus Delle Chiaje, 1830	13.33	3.33					
Sigalionidae sp.			3.33				
Spio sp. 1			3.33				
Spio sp. 2	3.33						
Spio sp. 3					13.33	10.00	3.33
Spionidae sp. 3	10.00						
Spionidae sp. 4			3.33			3.33	
Sternaspis scutata (Ranzani, 1817)					3.33	6.67	
Streblospio shrubsolii (Buchanan, 1890)	13.33						3.33
Terebellides stroemii Sars, 1835			3.33				
**Scaphopoda**							
Fustiaria rubescens (Deshayes, 1825)					3.33		

Table 4. Macrobenthic species identified in the samples collected in June 2024 from stations located at depths between 10 and 200 m. The values of their average densities from the three replicate samples, are expressed in individuals per square meter (ind./m²).

Species	10 m	20 m	30 m	40 m	50 m	100 m	200 m
**Bivalvia**							
Dosinia lupinus (Linnaeus, 1758)	3.33						
Gouldia minima (Montagu, 1803)		3.33					
Loripinus fragilis (R. A. Philippi, 1836)	6.67	16.67					
Lucinella divaricata (Linnaeus, 1758)	216.67						
Nucula nucleus (Linnaeus, 1758)				20.00	10.00		6.67
Parvicardium exiguum (Gmelin, 1791)	3.33	3.33					
Pitar rudis (Poli, 1795)	10.00						
Smaragdia viridis (Linnaeus, 1758)	3.33						
Tellina sp.	3.33						
**Gastropoda**							
Euspira nitida (Donovan, 1803)		3.33					
Phylliroe sp.						10.00	3.33
**Malacostraca**							
<u>Amphipoda</u>							
Ampelisca sp. 2				3.33			
Ampelisca sp. 3			3.33				
Aora sp.	30.00				6.67		
Dexamine spinosa (Montagu, 1813)		6.67					
Lembos sp.	10.00				13.33		
Perioculodes longimanus (Spence Bate & Westwood, 1868)	3.33						
<u>Cumacea</u>							
Campylaspis rostrata Calman, 1905	3.33						
<u>Decapoda</u>							
Alpheus sp.			3.33	3.33	3.33		
Anapagurus bicorniger A. Milne-Edwards & Bouvier, 1892			3.33	3.33	3.33	6.67	
Athanas nitescens (Leach, 1814)		6.67					
Callianassa sp.	6.67	6.67			3.33	6.67	
Ebalia cranchii Leach, 1817		3.33					
Galathea intermedia Lilljeborg, 1851					3.33		

Goneplax rhomboides (Linnaeus, 1758)		3.33				
Gourretia denticulata (Lutze, 1937)		3.33	3.33			
Pagurus sp.2			16.67	3.33	6.67	6.67
Polybius maculatus (Risso, 1827)					3.33	
Polybius navigator (Herbst, 1794)					3.33	
Processa sp.			3.33			
Upogebia tipica (Nardo, 1869)	10.00		33.33	16.67		3.33
<u>Mysida</u>						
Haplostylus sp.			3.33			
<u>Tanaidacea</u>						
Apseudidae sp.	33.33					
Chondrochelia savignyi (Kroyer, 1842)	3.33					
**Polychaeta**						
Abyssoninoe bidentata D'Alessandro, Cosentino, Giacobbe, Andaloro & Romeo, 2014			3.33			
Aphelochaeta marioni (Saint-Joseph, 1894)	3.33	6.67	3.33	10.00		3.33
Aponuphis brementi (Fauvel, 1916)			10.00	3.33		
Aricidea (Acmira) simonae Laubier & Ramos, 1974	3.33					
Capitella capitata (Fabricius, 1780)		3.33	13.33			
Capitella minima Langerhans, 1880	3.33	6.67				
Capitellidae sp. 3				3.33		
Chaetozone zetlandica McIntosh, 1911	3.33		6.67	3.33		
Cirratulidae sp.				3.33		
Diplocirrus capensis Day, 1961		3.33				
Drilonereis filum (Claparède, 1868)			3.33	3.33	3.33	
Euclymene oerstedii (Claparède, 1863)	6.67					
Euclymene sp.	10.00					
Glycera alba (O.F. Müller, 1776)		3.33			3.33	
Glycera fallax Quatrefages, 1850			3.33	3.33		
Glycera rouxii Audouin & Milne Edwards, 1833					3.33	
Glycera sp.3				3.33		
Glycera unicornis Lamarck, 1818	10.00					3.33
Glyceridae sp.2				3.33		
Kirkegaardia heterochaeta (Laubier, 1961)		13.33	6.67	10.00		
Levinsenia gracilis (Tauber, 1879)			3.33			

Lumbrineridae sp.2		3.33	3.33			
Lumbrineris latreilli Audouin & Milne Edwards, 1833			6.67	6.67		
Lysidice unicornis (Grube, 1840)		36.67	3.33			
Macroclymene santanderensis (Rioja, 1917)	6.67		3.33			
Magelona alleni Wilson, 1958		3.33				
Mediomastus capensis Day, 1961			3.33	3.33		
Mediomastus fragilis Rasmussen, 1973			20.00			
Nephtys hombergii Savigny in Lamarck, 1818	3.33					
Nereis zonata Malmgren, 1867	6.67					
Notomastus formianus Eisig, 1887		3.33	6.67			
Palola siciliensis (Grube, 1840)		3.33				
Paucibranchia bellii (Audouin & Milne Edwards, 1833)		3.33				
Petaloproctus terricolus Quatrefages, 1866		3.33	6.67			
Pilargis verrucosa Saint-Joseph, 1899			3.33			
Praxillella gracilis (M. Sars, 1861)	3.33					
Pseudoleiocapitella fauveli Harmelin, 1964	3.33		16.67			
Sabellidae sp.2	3.33					
Sigalion sp.2						3.33
Spio sp.4	10.00					
Streblospio shrubsolii (Buchanan, 1890)	23.33					
Syllis variegata Grube, 1860			3.33			
Terebellidae sp.		3.33				
Vermiliopsis sp.			3.33			
